# Book Reviews

**Published:** 1820-08

**Authors:** 


### CRITICAL ANALYSIS

OF

RECENT PUBLICATIONS, IN THE DIFFERENT BRANCHES OF MEDICINE AND SURGERY;

SELECT MEMOIRS, AND HISTORIES OF CASES;

### In the Literature of Foreign Nations.

---

Παῖρίδες ἄρα
Ανδράσιν, ὦ παῖραῖς ἄνδρες, ἀγαλλόμεθα.

---

*Medico-Chirurgical Annuary of the Civil Hospitals and Infirmaries of Paris; or, a Collection of Memoirs and Observations by the Physicians and Surgeons of those Establishments.* 4to. pp. 636; with fourteen Plates. Paris, 1819.

[In continuation from p. 80.]

THE concluding memoir in this volume is on *the Mental Alienation of Puerperal Women and Nurses;* by M. ESQUIROL, Physician-in-ordinary to the Salpetriere.

The author does not comprise under the above term either the temporary delirium which sometimes attends labour, or that occurring during *milk-fever*, or a sort of phrensy which appears to occur occasionally in young girls immediately after giving birth to bastard children in solitude. It is applied only to the chronic mental alienation which attacks women either soon after labour, during the time of lactation, or just after weaning.

This affection is of very frequent occurrence in France, in comparison with insanity from other causes; it ordinarily forms about one-twelfth of the whole number of lunatic women admitted into the Salpetriere, and in some years the proportion has been one-tenth. Thus, of eleven hundred and nineteen admitted during the years 1811, 1812, 1813, and 1814, ninety-two were cases of the kind above mentioned. It is of still more frequent occurrence amongst the rich, M. Esquirol states: in his private practice, the proportion is about one-seventh.

Of the ninety-two cases above alluded to, seventeen happened on the fifteenth and sixteenth days after delivery, (the extreme term of the flow of the lochiæ;) nineteen, from the second to the twelfth month, during lactation; nineteen immediately after weaning, either forced or voluntary: from these facts it may be concluded, the author observes, "1°, that these affections are more frequent in women in child-bed than in nurses; 2°, that the danger of their occurrence diminishes in a direct ratio to the remoteness from the time of delivery; 3°, that nurses are much more exposed to them after weaning, than during lactation."

Mental alienation as a consequence of labour, is sometimes announced by alarming presentiments, even during pregnancy; and sadness, from trivial or exaggerated causes, also preludes the development of the delirium: sometimes, however, the insanity appears suddenly. At the commencement, the patient appears to be in a febrile state; the skin is supple and moist; the face pale; the tongue white; the breasts shrunken; the abdomen is neither tense nor painful; sometimes there is very acute pain about the uterus; the pulse is small, weak, and concentrated; and there is at the same time either delirium in respect only to certain subjects, or *monomania*, more frequently *mania*, and rarely *demency*. Sometimes the most profound stupor precedes phrenitis, with which the puerperal mania may be confounded; but the head-ach, redness of the eyes, aridity of the skin, ringing in the ears, variation in the pulse, subsultus tendinum, and the violence and rapid increase of the symptoms, distinguish the two maladies.

Cases of mental alienation which are manifested during or after lactation, the author states, present but little difference, in respect to their character and progress, from the alienation which occurs under common circumstances: yet, he says, the "*facies*" has something peculiar, which enables us to recognize them, when we have been accustomed to observe patients of these affections.

Of the ninety-two cases above referred to, eight suffered dementy, thirty-nine melancholy or monomania, and forty-nine mania. Twenty-two of the patients were from 20 to 25 years old, forty-one from 25 to 30, sixteen from 30 to 35, eleven from 35 to 40, and two of 43. Thus the age at which it most commonly happens is from 25 to 30, which is the period of life at which mental alienation, considered generally, is most frequent.

The causes which especially dispose lying-in women and nurses to this affection, are, according to the author:

"Heredity, an extreme susceptibility, accessions of insanity anterior to any instance of pregnancy, accessions consequent on preceding labours or during lactation. In some cases, the predisposing causes are sufficient, not only to cause a paroxysm of delirium, but even to produce real lunacy: such are laborious parturition, the mere recurrence of labour or of lactation; the same physical circumstances bringing on the same moral and intellectual disorders. What is singular is, that women have been known who have become insane after having given birth to a male infant, and have remained free from such an accident after having produced a female. Some have been seen, in whom the delirium has manifested itself only after every second delivery; and others, who fell into the same state at the third or at the fifth month of lactation, without any assignable new exciting cause.

"The exciting causes are improprieties of regimen and moral affections.

"The impression of cold, in whatever manner it is applied, is, of all the improprieties of regimen, the most to be feared. Exposure to cool air, the application of cold water, whether the patient be exposed to currents of wind, or walk about in the open air, or place her limbs in cold water; cutting off the hair; and the abuse of heating medicines, by suppressing the lochiæ, provoke this affection. In the ninety-two women above noticed, it was excited in fourteen instances by physical causes; and the impression of cold produced the disease in ten out of these fourteen cases.

"Sudden weaning, either forced or voluntary, is not a rare cause of lunacy, if nurses neglect the precautions which prudence and experience inculcate. Thus, of the ninety-two cases, nineteen occurred immediately, or within a few days, after weaning. But this affection is very rare on the same occasion in the higher classes of society.

"Moral affections are the most frequent cause of the mental alienation of lying-in women and nurses; they are in regard to physical causes, in respect to frequency, as four to one. The deleterious and general influence of these causes has been appreciated in all ages. At Rome, they suspended a crown at the door of the house of lying-in women, to make it known that their habitation was a sacred asylum.

There exists a law at Haarlem, which orders that a sign shall be affixed to the houses on this occasion: this sign serves as a protection against sheriffs' and police officers, who might proceed to enter it for the execution of their official duties. What happens as a consequence of child-birth occurs also during and after lactation, but with less danger. Of ninety-two cases, forty-six ensued from moral affections. The fear of suffering it after having had a former attack, the despair caused by the loss of the infant or the desertion of its father, anger, terror, grief, domestic troubles, are so many exciting causes. The effects of terror were remarkable in 1814; since, of thirteen women who were admitted into the hospital, and had become insane after child-birth from moral affections, eleven were thus affected from terror: it was the same in 1815.

"The predisposing causes prepare, in a manner, lying-in women and nurses for the action of the exciting causes; both have more energy, in consequence of parturition and lactation exalting the susceptibility and mobility of women, and thus rendering them more accessible to surrounding and accidental influences. The latter, and amongst these moral affections especially, are more deleterious, in proportion to the suddenness of their action."

We have considered the preceding paragraphs as worthy of transcription in detail: of the rest of the memoir we shall give only an abstract.

When the physical and moral agents above designated exert their influence on a lying-in woman, the lochiæ diminish or become absent, the milk does not appear in the breasts, or these organs sink and become lax, and then the mental alienation is evinced. In some cases, which are however very rare, the lochiæ are freely evacuated, and have even an hemorrhagic character. It is the same in respect to the milk: sometimes there is no suppression of it, though it becomes less abundant; it has not its proper nutritive and wholesome qualities, and the infant refuses to suck; and in other cases the alienation is developed when there is no diminution of milk, and lactation is continued with benefit to the infant.

In reply to the question, "Is the suppression or diminution of the secretion of milk the cause or the effect of the mental alienation?" M. Esquirol only states, that "it is true that insanity is most commonly manifested in women who do not nurse their children. Of our ninety-two patients, twenty-nine were unmarried women: now these women but rarely nurse their infants, and there is proportionally more married women who give birth to children. The greater number of facts prove that the milk diminishes, is suppressed, or is altered in its qualities, before the explosion of the delirium; but there are many which also show that its suppression or diminution has not taken place till after the appearance of mania or melancholy."

M. Esquirol seems to have forgotten, in this calculation, that unmarried women are much more frequently exposed to the influence of the moral affections qualified to produce the malady, than those who are married; and, therefore, that the great proportion of the former, in his list of patients, does not at all tend to prove that the want of lactation is a cause of the disease.

Some useless discussion, chiefly founded on observations made by some of the older anatomists who did not know the difference between milk and purulent matter, follows in the memoir of M. Esquirol, after the question whether the milk acts as a foreign body, in the accidents consequent on parturition and lactation, on the brain, and other organs. A report of the author's observations respecting the results of the disease, is then adduced. Mental alienation consequent on parturition, he says, is generally cured. If the predisposition to it is not very great, more than half the cases of it terminate in health. Fifty-five of the ninety-two had this result at La Salpetriere. They terminated by the re-establishment of the lochiæ, the appearance of milk in the breasts, abundant leucorrhœa, mucous alvine dejections, the return of menstruation, subcutaneous abscesses, and, in a very few instances, during pregnancy. The duration of the disease was variable: of the fifty-five just noticed, it terminated in four cases in the first month, in seven in the second, in six in the third, in seven in the fourth, in five in the fifth, in nine in the sixth, "in seventeen in *the following months*, in two after two years." The last statement is exactly in the terms of the author: we know not what period he intends to signify by "in the following months," and he has no-where explained it.

Only six of the ninety-two cases terminated in death: this event happened in one instance after the lapse of six months from delivery, in one after a year, in two after eighteen months, in one after three years, in one after five years.

Dissection of the bodies of lying-in women or nurses who had been insane for a greater or less length of time, showed nothing peculiar on rigorous consideration; nothing which was calculated to indicate the material cause of the malady, or to discover the seat of it.

After passing in review the principal of the means of cure which have been employed in different times, M. Esquirol states the results of his own experience respecting the proposed remedies. The recurrence of pregnancy, which has been supposed by many to be likely to remove the mental disorder, has not been found successful by M. Esquirol, "except when this affection was accidental, and did not depend on any severe anterior or predisposing cause." Bleeding, he says, should not be resorted to without some precaution. "Leeches to the vulva or to the thighs, when there are evident signs of plethora, and when the sanguine temperament predominates, are sometimes useful. Cupping, vesicatories, sinapisms, applied at one time to the legs or the thighs, at another to the nape of the neck, with a mild sudorific or purgative drink, according to the tendency of nature, should be preferred to too-energetic means. Some have been cured, soon after delivery, by purgative glysters; they have been used three times a-day, with a mild purgative tisan: the patients were at the same time submitted to a severe regimen, and ate but little. Emetics, repeated several times in quick succession, have also been successful in subjects of a very lymphatic habit. It sometimes happens that vesicatories, which have not succeeded at the onset of the malady, in its stage of irritation, have produced better effects when repeated at the lapse of some time from its invasion. Tepid-baths, especially the hip-bath, and sometimes hot-bathing, wonderfully aid the other curative means when the disease is chronic. When it has persisted long, especially in nurses, and after the use of evacuants, benefit is sometimes derived from leeches applied to the vulva, emmenagogues, and other medicines, calculated to produce the menstrual evacuation. It is hardly necessary to remark, that these patients should be submitted to the same general principles of treatment as other lunatics; that isolation, hygienic aid, and moral means, should not be neglected, and that they have alone been sufficient to effect a cure, although more rarely than in other cases of insanity."

We have deemed it prudent to transcribe the whole of the author's remarks on the treatment, although they present nothing of much peculiar importance, since it is desirable to know what a man of his talents and experience has to say respecting so serious a malady.

---

*The Natural History of the Medicines, Aliments, and Poisons, taken from the Kingdoms of Nature.* By J. J. Virey, Doctor in Medicine of the Faculty of Paris, Member of several Learned Societies, Professor of Natural History at the Atheneum at Paris, Master in Pharmacy, late Pharmaceutist-in-chief to the Military Hospital of the Val-de-Grace, Associate and Correspondent of several Foreign Academies, &c. 8vo. pp. 570. Remont and Ferra, Paris, 1820.*

This work, like most of the writings of M. Virey, displays great erudition and much original observation: these are the things most essential to a treatise on a branch of natural history. Great depth of judgment is not of the first importance in the

---

* *Histoire Naturelle des Médicamens, des Alimens, et des Poisons, tirés des trois Règnes de la Nature, &c.*

compilation; if the materials are arranged with tolerable perspicuity, the reader may supply what has been deficient in the author in this respect. Hence we may peruse even the writings of BURMANN on this subject with advantage. That before us will be read with considerable pleasure. M. Virey has here had no occasion nor opportunity for introducing his Platonic and Plotinian reveries about the mind, or to express his concurrence in opinion with the honest SAINT AUGUSTIN, that many diseases of the human body are produced by devils; or, forgetting the due gravity of the medical character, to dance a hornpipe through all the mystic regions disclosed by Jamblicus or Apollonius. None of these things is to be found in this book. The author has employed his talents in a laudable manner, and applied them to a good purpose.

The object of the work is to give a general and complete account of the natural history of all the substances which are now, or have been, used in medicine; in which those substances are classed and arranged according to their analogical relations in respect to their affinities, structure, or organization; and the author's intention has been fulfilled in an able manner. The first part commences with an introduction, comprising some general views respecting the properties and qualities of medicines, with reflections tending to prove the necessity of the knowledge of their natural history to the physician; and some curious and very interesting remarks on the important indications which the colour, smell, and taste, furnish of the properties of vegetable substances, in addition to those which are pointed out by specific analogies according to the systems of botanists.

Men in all ages have endeavoured to outstrip the slow progress of experimental enquiry, in obtaining knowledge of the medicinal qualities of the multitude of plants dispersed over the globe, and various suppositious or imperfect means of effecting this have at different times been imagined. During the reign of astrological superstition, each plant was supposed to be governed by a certain planet, which was supposed to preside over the functions of a certain part of the body; and hence the plant was considered to be useful in the diseases of the part, according to the influence of the planet. As these reveries subsided, equally ridiculous notions, founded on the resemblance of the colour of certain plants to that of certain parts of the body, or to the skin in particular diseases, or on an imaginary resemblance in their figure to some of our organs, became prevalent: many curious instances of which are related by Dr. PARIS, in his Pharmacology, when treating of the doctrine of *signatures*. After the favourers of those notions came the chemists, who could not imagine that there was any better way to discover the virtues of plants than to put them in an alembic, and distil and torture them by fire; and, when they had extracted from them their *empyreumatic oil*, their acid *spirit*, their *phlegm*, their salts, and found the *caput mortuum*, they believed they had unveiled all the wonders of the vegetable creation. But, after having made nearly two thousand similar analyses, and having found what was poisonous furnished almost exactly the same products as what was good for food, so that there was not the least difference between bread and hemlock, they were obliged to acknowledge the complete inutility of this method, to distinguish the qualities of plants whose effects on the human body are so opposite. The modern chemists certainly proceed in a more rational manner, by separating the component parts of vegetables without alteration of their immediate products. But it is not probable that this method will ever be sufficient to direct with much certainty, or to much extent, the researches of the physician. That which has hitherto been most extensively useful in this respect, has been the analogies which have been assumed by botanists for the classification of this part of organized nature, and especially the method of Jussieu. But this method is not universally a safe guide for our conduct; as we exemplified in a few instances in the work of Dr. Paris: besides the positive differences in the qualities of some plants of the same species, under all circumstances, many others arise from variations in respect to the climate and soil in which they grow. Dr. Paris, in speaking of the umbelliferæ, says, "those which grow on dry ground are aromatic, whilst the aquatic species are among the most deadly poisons;" a remark which M. Virey extends to the ranunculacea, pediculares, joncacea, aroides, and the cruciferes. And he also states that many vernal plants acquire acrid properties when they grow in water, as the daphne, draba, and chrysoplenium, and lose those properties when cultivated on dry ground. We, in our review of the Pharmacologia, had mentioned celery as an exception to the law above mentioned; but M. Virey says that this plant even becomes poisonous if it grows in water. Others only develop their virulent qualities in a hot and dry soil, or have them only in their full power, as the strychnos, the euphorbiacea. Different parts of plants also have different qualities; and, in the cultivated potato even, the stalk and berries approach somewhat towards the virulence which characterize the greater part of the solanums. The researches of modern chemists have shown that the poisonous principle of plants may generally be wholly extracted from them in the form of an alkaline substance; and it is an interesting fact in regard to this discovery, that, according to M. Virey, "acid vegetables are never poisonous." Those poisonous plants, too, which have acid fruits or other parts, commonly lose their poisonous qualities in those parts, as the lycopersicon, the pulp of the fruits of the strychnos, the garcinia, the averrhoa, which are innoxious; whilst the bark, and other parts of those trees, are more or less poisonous. The poisonous qualities of many plants exist only in their odorous and sapid parts, which may be removed by heat or washing them in water. Thus, the fecula of the arum, briony, and the mandragora, loses its virulence on being washed, and becomes a wholesome nutritious substance. It is only necessary to boil the root of the manioc to obtain the useful aliment cassava.

"Every plant," says M. Virey, "every substance, of an agreeable taste or odour, is ordinarily useful to our body in the state of health; all those which are of a disagreeable smell or taste, are noxious and repugnant to us; all those which excite nausea, are either emetic or poisonous. Here are principles by which all animals are governed in the search after their food; and the savage as well as the ape is guided solely by his senses."

The author's disquisition on this subject is one of the most curious parts of his work; and we shall endeavour to give the reader some idea of it in an analytical abstract. Let us, however, first remark, that the objections which may be brought against the statement made in the foregoing paragraph, from the conduct of civilized persons, are not well founded: children may swallow the berries of the mezereon or of the belladonna; and we may be deceived by the beautiful form and colour, the citron-like odour, and the mild taste, of the fruit of the hippomane, and be induced to eat it when it has become poisonous; but these errors depend on the comparatively dormant state of our senses, and the submission of our instincts to our powers of reasoning, in the mode in which we are educated, not on a natural inability in man to distinguish in the vegetable productions of nature what is beneficial from what is hurtful to him.

Several botanists, and Linnæus in particular, rejected from the number of the characters of plants the designation of the colours of their flowers, or other parts, as too variable to be described with them. As a general principle, this proscription is founded on a multitude of facts: nothing is more frequent than changes in the colours of the flowers of the poppy, tulip, the ranunculus, the alcea, the anemony, hyacinth, the delphinium, the auricula ursi, the balsams, and a thousand others, which are altered at will under the hands of an able gardener. Many varieties in this respect depend also solely on soil and climate; but it is of importance, in regard to medicine, to understand that this change of the colour of the flower or other parts of a vegetable, presents the character of some predominant principle in it: with the change of colour, this principle, or the medicinal quality which it possesses, also changes in equal proportion. Thus the red and white poppy, the red and damask rose, have very different medicinal qualities. It is also true, that the deeper the hue of a vegetable becomes, the greater will be the degree of intensity of the property allied with it. This is well known by the effects of etiolation in plants used on our tables as food. This effect is produced, in a certain degree, by cultivation in a very rich soil; and hence the medicinal plants of our gardens are much less efficacious than those of the same species which have grown wild on comparatively barren ground. Linnæus, however, only excluded the colours of plants from the list of their characters in a botanical view: he was aware of the utility of indications from this source in regard to their medicinal qualities, and indeed stated that these might generally be known from their colour, taste, and smell; an assertion which is proved to be true to a great extent by the disquisition of M. Virey.

White flowers, M. Virey says, are in general those which least of all preserve their properties by desiccation; they are mostly of a very aqueous nature. White is the most frequent colour amongst the flowers of cold countries, in the alpine plants, and in those which bloom before the sun has acquired much power. As this colour is the indication of weakness and of superabundant humidity, it excludes, with a few exceptions, tonic and astringent properties, and much strength of taste and odour. One exception exists in regard to the white flowers of the cruciform plants, which are more acrid than the yellow or other coloured flowers of this class. The white woods, as the betula, willow, poplar, fir, &c. are also abundant in cold and moist countries; but all these vegetables contain but little of the astringent and tonic principles which abound in the red or brown woods of warmer climates, as the oak, chesnut, the dyeing woods, the acajou, the tec, the cedar, the guaiacum, ironwood, ebony, &c. The white and soft woods of southern countries, as the bombax, the maple, and other malvaceous trees, are devoid of active properties. It is then evident that, in general, the white colour is not allied with the more stimulant medical qualities: thus the white squill is less acrid than the red; the white dianthus has less aromatic property than the red; the colourless gums and resins are the most pure and insipid; the white canella, the pale cinchona, the white sanderswood, have much less active properties than the more coloured species, &c.

The yellow colour is the one most diffused in the vegetable kingdom, and, though often combined with very different principles, it is hardly ever found with acids; it is, on the other hand, the most constant index of the bitter principle. If we review the whole of bitter vegetable substances, there will be found but few which are not yellow, or which do not impart dye more or less deep of this colour. Thus, the juice of the aloe, chelidony, rhubarb, gentian, the flowers of broom, colutea, lygeum, iris, all the chicoracea, the stalks with bitter yellow juices of the corymbiferes, arnica, calendula, doronicum, tagetes, chrysocoma, solidago, jacobœa, conyza, eupatorium; all the bitter aromatics, as the wormwoods and artemisia, the abrontona, santolina, tanacetum, chamomile, matricaria, chrysanthemum, and many others, present this combination of the yellow colour with the bitter principle. We find also bitter yellow juices when we cut open many of the cynarocephales, as the artichoke, the carthamus, the serratula, the jacea, the centaurea, and carduus, the yellow and bitter wood of the barbary. There are very acrid and lethiferous bitter yellow juices in the oluanthes, the phellandria, in the flowers of several ranunculacea, of the trollius, argemone; others are violently purgative, as the gamboge. Not only the substances naturally yellow, but even those formed by art, as the *bitter* of Welter, and the other yellow products obtained by the action of nitric acid on organic bodies, have this taste; it is also found in the bile of animals; so that this modification of colour seems commonly to assume the same properties. Most of the bitter barks and woods are also of a more or less clearly marked yellow colour. There are, however, many exceptions to the principle thus illustrated by M. Virey; as in the carrot, turmeric, and liquorice-root, water-trefoil, and many of the mild and dulcid leguminous plants are of a more or less yellow colour: thus, besides the common liquorice, there is the gleditsia triacanthos of Linnæus, the arachis hypogola, the astragalus glycyphillos, several of the species cystisus, and the hedysarum, the galega, lathyrus tuberosus, the robinia pseudo-acacia; and some seeds, as those of mustard.

The red colour in vegetables is almost the universal character of acidity and astringency: acids, it may be remarked, have the power of turning red many shades of colour in vegetables, especially blue. Hence flowers pass so frequently from blue to red, and from red to blue, according as acidity and alkalinity predominate. Such is the case with the flowers of the borrage, bugloss, scabious, polygala, echium, pulmonaria, aquilegia, delphinium, marchantia, cyanus, &c. There is no red fruit whatever that does not owe to some acid the manifestation of this colour. This is familiarly known in cherries, gooseberries, strawberries, raspberries, barberries, mulberries, tamarinds, ananas, and a multitude of others. If, however, the acid of any of those fruits be saturated by an alkali, a blue or greenish colour is produced; and, in proportion as those fruits ripen and lose their acidity, they will generally be found to assume a bluish hue. The insects which produce red dyes often draw them from acid or astringent plants, as the cochineal from the cactus opuntia, the fruit of which is red and acid; the kermes from an oak which contains an abundance of tannin; the coccus polonicus from the scleranthus perennis, a red and astringent herb. The red colour of the gum-lac is also owing to the acidity of the insects which form it on diverse vegetables. An acid or astringent principle exists in the whole family of the rosacea (of Jussieu); and all the family of the myrtoides are astringent, presenting red juices which abound with tannin. It is the same with the calycanthemes. There seems to exist no red vegetable matters which are not acid or astringent, though there are some acid plants which are not red.

The reddish-brown colour is the constant sign of tonic and astringent properties, and it is principally observed in the ligneous parts of vegetables, as the wood, the bark, and root. Those which are most red are the most astringent. The astringent principle seems to be so inherent in this modification of colour, that they are, perhaps, never found separate. All the substances which are used for the process of tanning are of this hue; as the oak-bark, used in England, France, and the United States; the capsules of the Indian chesnut, used in Asia Minor; the prunica, at Tunis; the acacia, in Egypt; the myrtle, in Naples and the Illyrian provinces; the coriaria and rhus cotina, about Montpellier; the rhus is also used in Hungary; the uva ursi, in Sweden; the fir, in Norway; the willow-bark, in Westrobothnia; the betula, in Lapland; the tormentil, in the Feröe Islands; the sumach, in Albania and Macedonia. This bitter and astringent principle is sometimes combined with an aromatic oil, as in the barks of canella, cassia lignea, several of the species laurus, sassafras, pimento, cherianthus, zedoary, galanga, cypress, the Arabian costus, and other aromatic substances of a reddish-brown colour. Vegetable juices which contain it soon become brown on being exposed to the air and light, as the sap of the amentaceous trees, the pulp of apples, pears, &c. and even several woods.

The green colour is not so precisely allied to any particular property as the foregoing. It is, in general, accompanied with an acerb or styptic taste. All fruits manifest this taste before their maturity. Those fruits which always remain green, as the olive and buckthorn, preserve a strong styptic taste. The pistacia, notwithstanding the emulsion it forms, retains an austere taste. The fruit of the wild plum and the crab-tree, which have a greenish pulp, are very acerb. The same qualities are generally found in the leaves of vegetables that are of a deep-green colour. This acerb principle is found combined with the bitter in wormwood, southernwood, balm, &c. plants which give an intense green colour mingled with yellow to sulphuric ether. We find too that, as green leaves of plants begin to decay and become bitter, they pass to the yellow colour; the same may be observed with respect to fruits.

The blue colour, as already indicated, is generally allied with an alkaline principle, as the red is with the acid. Vegetable substances of the latter colour are but rarely poisonous; but those which are blue cannot, in general, be safely eaten, although this statement cannot be made without many exceptions. Thus, the aconites, the wild delphinia, the mandragora, several of the solanums, the convolvuli and apocynia, the vinca, the lobelia, and the phytemna, the enarchantia, pulsatilla, clematites, and several of the leguminous tribe, have blue flowers, always accompanied with acrid or unwholesome principles. Those flowers in which the blue passes into red or white, are devoid of these principles; it is those in which the blue colour is fixed and intense in degree, and as it were essential to the plant, that the deleterious qualities are found. Some vegetables, without having blue flowers, have their foliage of a bluish hue, which is the sign of more or less hurtful qualities. Thus, the papaveracea, the argemone, the aquilegia, the gratiola, and plumbago, the clematites, the lactuca virosa; the poisonous umbelliferæ, as the œnanthe and phellandria: some of the varieties of hellebore, the cynanchum, and of the euphorbia, and many other plants, present many examples of it. It is worthy of remark, too, that when the lettuce is altered by cultivation, it in a great degree loses its bluish colour with its deleterious principles. It is necessary to distinguish from the blue colour that which is produced by a fine whitish down on many leaves and fruits, which appears blue though it is not so, and is easily removed, and which does not indicate any unwholesome qualities. Many of the marine alkaline plants are of a bluish colour. Those champignons which present a bluish juice on being broken open, are acrid and deleterious; such are several of the amanites. Thus, the blue colour generally indicates alkaline properties, and more or less deleterious qualities; while those plants of a violet hue, in which the red predominates over the blue, are generally devoid of them.

The black hue is but rarely seen in the petals of plants; it is most common in the bark which envelops their roots, and in the husks of their seeds. This colour generally indicates deleterious qualities. The brown flowers, verging to black, of the belladonna, hyosciamus, of several of the solanums, the cynoglossa, scrofularia, and aristolochia, black hellebore, &c. contain a nauseous principle more or less acrid and deleterious. The more lethiferous of the solanums have a blackish foliage, indicative of their qualities. It may be remarked, too, that in those plants whose foliage is spotted with black, as the arum, the spotted ranunculacea, the spotted persicaria, the geranium, hircinum, the spotted hemlock, the ivy, the galeobdolon, the black poppy, the aconites, &c. there exist principles more or less deleterious, which are not found in the neighbouring species. These indications are still more striking in the colours of fruits; for there does not, perhaps, exist one of a black hue which can be eaten without danger; and this assertion is equally true in respect to the juices of vegetables.

The author thinks it may then be concluded, from what is indicated in the foregoing observations, that, with some few exceptions, the colours of vegetables indicate generally their predominant principles, and that they may serve to establish differences in them in the materia medica. Thus, as a summary, it may be stated, that

The white hue indicates emollient and nutritive qualities;

The yellow, bitter, purgative, and stimulant, qualities;

The red, acid properties and astringent qualities;

The reddish-brown, the tanning principle and tonic qualities;

The green, an acerb, sour, styptic, and astringent, principle;

The blue, acrid, alkaline, and caustic, properties;

The black, nauseous, deleterious, stupifying, qualities, acting especially on the nervous system.

These propositions are, we think, true to a certain extent, and sufficiently so to furnish some useful indications for general views; but the exceptions are nevertheless very numerous, as every person may prove by walking through a botanic garden, for the purpose of counterbalancing the above observations in their favour by others which are adverse to them.

---

